# Molecular data storage using direct analysis in real time (DART) ionization mass spectrometry for decoding

**DOI:** 10.1038/s41598-023-43658-x

**Published:** 2023-10-03

**Authors:** Veronika Pardi-Tóth, Ákos Kuki, Marcell Árpád Kordován, Gergő Róth, Lajos Nagy, Miklós Zsuga, Tibor Nagy, Sándor Kéki

**Affiliations:** 1 Department of Applied Chemistry, Faculty of Science and Technology, University of Debrecen, Egyetem tér 1, Debrecen, 4032 Hungary; 2 Doctoral School of Chemistry, University of Debrecen, Egyetem tér 1, Debrecen, 4032 Hungary

**Keywords:** Chemistry, Materials science

## Abstract

Molecular data storage is becoming a viable alternative to traditional information storage systems. Here, we propose a method where the presence or absence of a given molecule in a mixture of compounds represents a bit of information. As a novel approach, direct analysis in real time (DART) ionization mass spectrometry is used to recover and decode the information stored at the molecular level. Nicotinic acid derivatives were synthesized and used as the ‘bit compounds’. Their volatility and ease of ionization make these molecules especially suitable for DART-MS detection. The application of DART-MS as a method with an ambient ionization technique, enables the re-reading of digital chemical codes embedded in the material of ordinary objects. Our method is designed to store and read back short pieces of digital information, up to several hundred bits. These codes can have the function of barcodes or QR codes, as shown in our proof-of-principle applications. First, modelling a QR code as a link to our university's website, three solutions were prepared, each representing 22 bits. Proceeding further, the bit compounds were incorporated into a polymer matrix that is suitable for 3D printing, and a toy ship was created with a hidden barcode. In addition, decoding software was developed to process the DART-MS spectra. The nicotinic acid components representing the bits dominated the DART-MS spectra and error-free decoding was achieved.

## Introduction

With the enormous growth in the amount of data produced by mankind, there is a growing global demand for digital data storage systems. However, the pace of development of traditional semiconductor-based technology is slowing down and, what is more, the data storage reliability of memory cards and magnetic disks tends to degrade over time, usually within a few decades. Therefore, attention is turning to the development of molecular-scale platforms for information storage. Inspired by living systems, the use of artificial DNA as an information-storing biopolymer is being intensively researched^1–4^. In addition to DNA, a variety of sequence-defined synthetic polymers have been developed in which the monomers represent the individual bits of digital information^2,5–7^. Tandem mass spectrometry (MS/MS) is the most commonly used method to access the information stored in these digital polymers. However, the synthesis and decoding of long macromolecular sequences are still challenging. To overcome these difficulties and simplify the methodology, mixtures of sequence-defined oligomers^8,9^ or small molecules are used for molecular data storage^10–15^. In this approach, the presence or absence of a given molecule in the mixture (and the presence or absence of the corresponding *m*/*z* peak in the mass spectrum during the read-out) encodes a bit of information. Typically, matrix-assisted laser desorption/ionization mass spectrometry (MALDI-MS) is used to decode the digital data, which is assembled and stored on a stainless steel MALDI target plate. However, using ambient ionization mass spectrometry (AIMS), such as direct analysis in real time (DART-MS)^16^ and desorption electrospray ionization (DESI-MS)^17^ mass spectrometry, it is possible to analyze the samples in their native state without any time-consuming sample preparation. As a result, AIMS methods are able to read the molecular information that is present or even hidden on the surface of common objects. Recently, Amalian and co-workers demonstrated the power of DESI-MS/MS in decoding of digital information from surfaces^8^. In addition to mass spectrometry, information stored at the molecular level can be read back by other methods, such as ^1^H-NMR^13,18,19^, fluorescence^20^, or Raman spectroscopy^21^, or gas chromatography^13^.

In this work, we took a novel approach by using DART-MS to recover and decode the information stored at the molecular level. Our aim was not primarily to increase the density of molecular data storage but to explore new ways of incorporating digital chemical codes into the material of ordinary objects, which can have the function of barcodes or QR codes.

## Experimental

### Chemicals

2-(3-pyridinyloxy)nicotinic acid, 5-(3-formyl-4-methoxyphenyl)nicotinic acid, 6-{[benzyl(methyl)amino]methyl)nicotinic acid, 4-amino-nicotinic acid ≥ 97% (HPLC), nicotinic acid ≥ 98% (starting materials) and 1-hexanol ≥ 98.0% a.r., 1-heptanol 98% a.r. were obtained from Sigma Aldrich (Taufkirchen, Germany). Methanol ≥ 99.9% (UHPLC-MS), ethanol 96% (v/v) a.r., 1-propanol ≥ 99.5% a.r., 1-butanol ≥ 99.5% a.r., 1-pentanol ≥ 98.5% a.r., sulfuric acid 96% a.r. were purchased from VWR (Debrecen, Hungary). Dichloromethane a.r. (stab. with amylene),* n*-hexane 96% a.r., ethyl-acetate a.r. wich were used for the purification, were obtained from Molar Chemicals (Halásztelek, Hungary).

Polypropylene Tipplen R 660 was purchased from MOL Petrolchemicals Plc. (Tiszaújváros, Hungary). Iron oxide pigments Bayferrox 130 M and Bayferrox 3910 obtained from Lanxess (Cologne, Germany).

### Synthesis

Chromatographic separations were performed using silica gel (Merck, 70–230 mesh). Thin-layer chromatography was carried out on Kieselgel 60 F254 (175–225 μm layer thickness, Merck). ^1^H NMR spectra were recorded with a Bruker AM 360 (360 MHz for ^1^H) CDCl_3_ solution unless otherwise specified (internal standard TMS, δ = 0 ppm).

Conc. H_2_SO_4_ (8.0 eq.) was added to a suspension of nicotinic acid derivatives (200 mg, 1.0 eq.) in *n*-alkyl-alcohol (3.4 mL) and the solution was stirred at reflux temperature by monitoring with TLC (hexane–ethyl acetate = 1:1, v/v). After completion, the reaction mixture was allowed to cool to room temperature, neutralized with solution of NaHCO_3_ (5 w%). The aqueous phase was extracted with dichloromethane (3 × 25 mL). The combined organic layers were dried (MgSO_4_), concentrated under reduced pressure and the residue was purified by column chromatography (hexane–ethyl acetate = 1:1, v/v) to give nicotinic acid alkyl ester.

The characterization of the nicotinic acid derivatives is detailed in the Supporting Information.

### Direct analysis in real time ionization mass spectrometry

The DART-MS measurements were carried out with a MicroTOF-Q type Qq-TOF MS instrument (Buker Daltoniks, Bremen, Germany), equipped by the DART SVP ion source (Ionsense, Inc., Saugus, MA, USA). All spectra were recorded by a digitizer at a sampling of 2 GHz. The spectra were evaluated by the DataAnalysis 3.4 software from Bruker.

The ion source temperature was 350 °C, He (5.0) was applied for the ionization. The samples were inserted manually to the source.

### Extrusion

A Composer 450 type extruder from 3Devo (Utrecht, the Netherlands) was used to make the 3D printable filament. This device contains 4 heating zones. The temperature profile was 205, 215, 205, and 202 °C (from hopper to nozzle). The screw speed was 4.3 RPM, and the nozzle diameter was 4 mm. In order to achieve a filament with a diameter of 1.75 mm, the puller speed was controlled automatically. The selected nicotine derivatives were dissolved in methanol. Then 21 g of polypropylene (PP) granules and 0.15 g of pigment were added to the solution. The mixture was homogenized and then the solvent was evaporated. The final concentrations of the nicotinic acid derivatives in the PP matrix are given in Supporting Information Table S1. The coated granules obtained as a product were fed into the hopper of the extruder. After the pulling, the system was cleaned with 300 g of PP granules to avoid mixing the key components.

### 3D printing

A Prusa MK3S + type printer (Prusa Research a.s., Praha, Czech Republic) was used for 3D printing. The nozzle diameter was 0.4 mm and the layer height was 0.2 mm. The nozzle temperature was set at 240 °C for the first layer, and 215 °C for the others. The bed temperature was constant at 40 °C. The bed was coated with adhesive PP tape.

## Results and discussion

**Figure 1:**
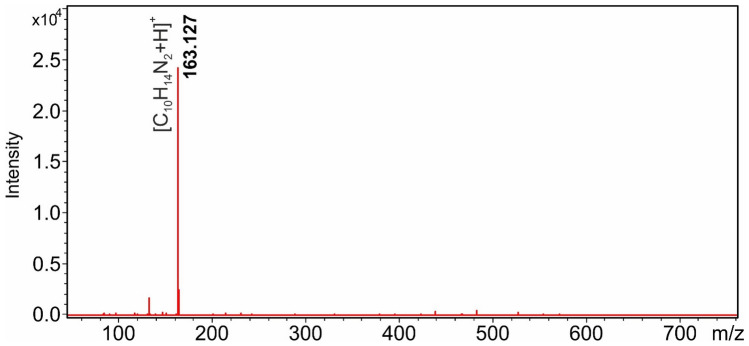
DART-MS spectrum of a piece of cloth previously exposed to cigarette smoke (subtracting the background spectrum of the DART source). ([C_10_H_14_N_2_ + H]^+^, protonated nicotine).

In a previous study, we found that DART-MS is particularly suitable for the detection of nicotine^22^. As shown in Fig. 1, nicotine adsorbed on the surface of various objects from air polluted with tobacco smoke can be detected with high sensitivity and intensity by the DART ion source.

**Scheme 1:**
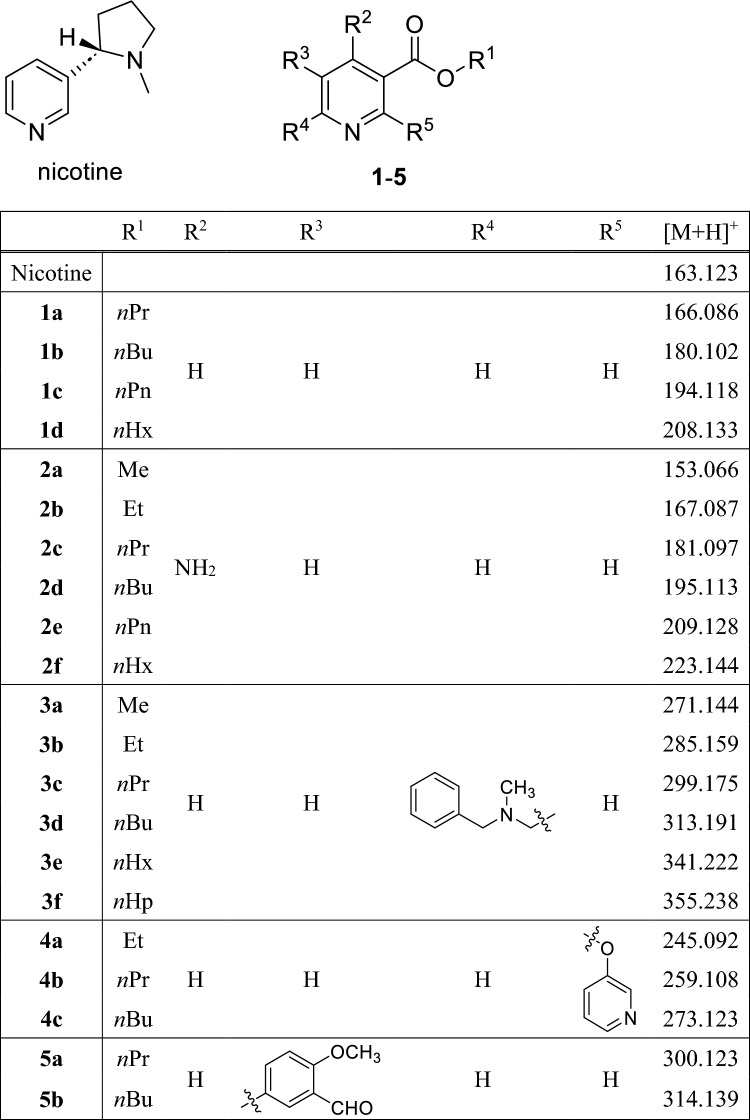
Chemical structures of the synthesized nicotinic acid derivatives and the *m/z* values of their protonated ions.

The protonated [M + H]^+^ ion of nicotine at *m*/*z* 163.127 dominates the mass spectrum as the base peak. Our assumption was that their volatility, thermal stability, and ease of ionization under DART conditions make nicotine and its derivatives highly suitable for molecular data storage in the case when DART-MS method is used for information retrieval. As a first step in our research project, we have synthesized a series of nicotinic acid derivatives, whose presence or absence will play the role of a digital bit in the mixture on the target surface and in the DART-MS spectrum when read back. The structures of the 22 synthesized derivatives are shown in Scheme 1.

In order to minimize or eliminate the bit error rate, i.e., the probability of a stored bit being decoded incorrectly, it is important that the components have approximately the same peak intensity in the DART mass spectrum.

**Figure 2:**
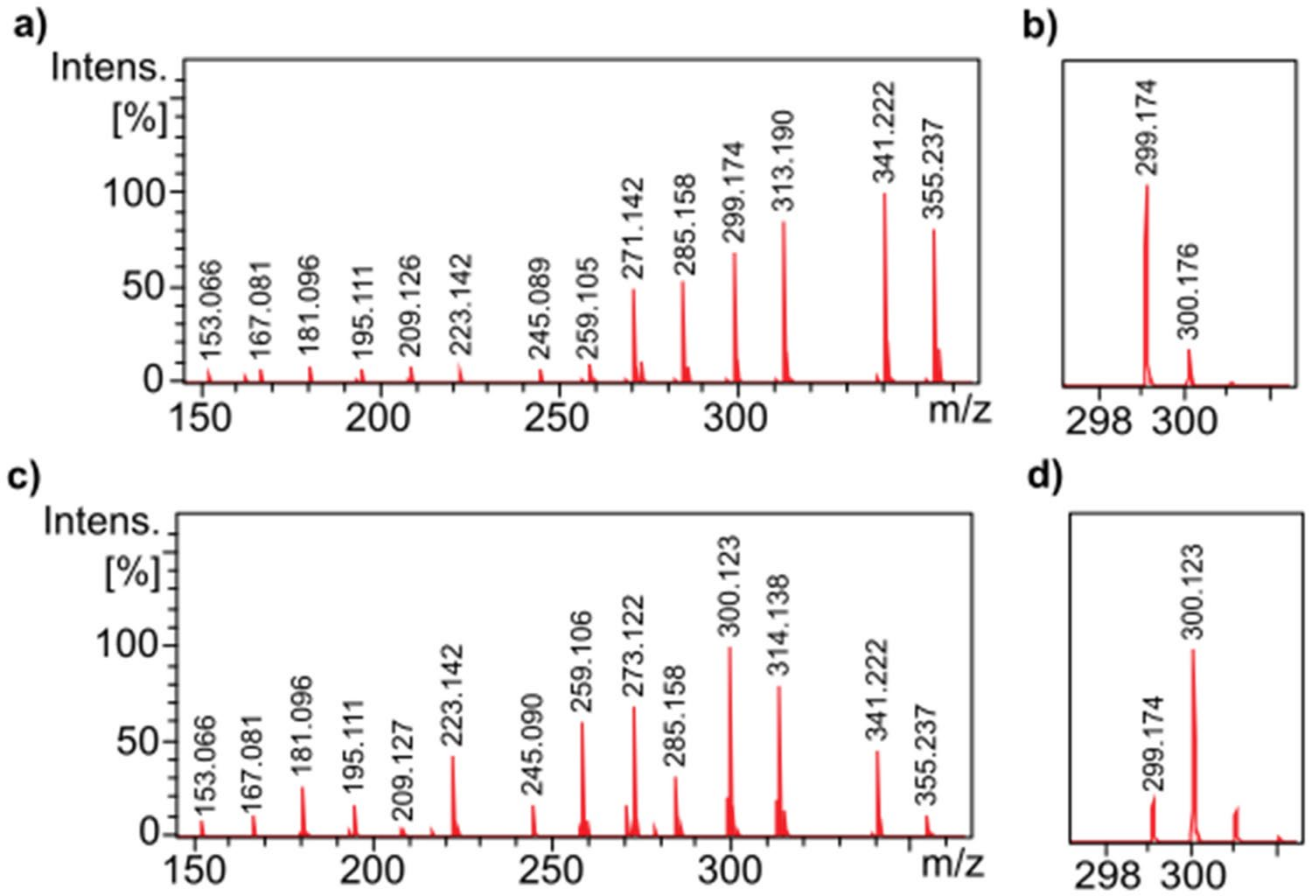
DART-MS spectrum of a solution containing all the 22 nicotinic acid derivatives (**a**, **b**) in the same concentration, (**c**, **d**) in the suggested concentrations. (The background spectrum of the DART-MS is subtracted).

Figure 2a shows the DART-MS spectrum of a solution containing all 22 nicotine derivatives at the same concentration (0.8 mg/mL). It can be seen that, although the components have similar structures, there are considerable differences in the intensities of the mass peaks due to the mass discriminant effect and/or the different ionization efficiencies. Another issue that may hinder decoding is that the monoisotopic peak of some derivatives overlaps with the first or second isotope peak of a component with one or two fewer m/z values. For example, as seen in Fig. 2b, it is difficult to decide whether the peak at *m*/*z* 300 belongs to compound **5a** or is the first isotopic peak of derivative **3c**. In order to overcome these difficulties, the concentrations of the derivatives were modified. First, the intensity ratios of the compounds were identified in a mixture with the same concentration (Fig. 2a). Based on these ratios, the concentrations were corrected in two iterative steps to obtain approximately similar intensities except for one or two *m/z* neighbors, where the larger components were overweighed. The suggested concentrations are given in Supporting Information Table S2 and the DART MS spectrum of the solution prepared by considering them is shown in Fig. 2c and d. As seen in Fig. 2d, the existence of compound **5a** at *m*/*z* 300 can be unambiguously determined.

In the following, we present two proof-of-principle applications of our novel chemical encoding method. In our first example, we modelled a QR code containing a link to our university's website. Three solutions were prepared, each representing 22 bits. The value of the *i*th bit of this 22-bit digital word is encoded by the presence or absence of the *i*th nicotine acid derivate, out of 22 (see Scheme 1) ordered by *m*/*z* value, in the mixture solution. The characters of the website URL were encoded using the alphanumeric mode of the ISO/IEC 18004 QR Code standard^23^. In this code system, the input data characters are divided into groups of two characters which are encoded to 11-bit binary codes. This means that with the three 22-bit words (three mixture solutions), we can encode 12 characters, 9 of which are used to encode the URL of our university's website ("UNIDEB.HU"). The DART sampling rods were immersed in the sample solutions and then inserted into the DART ion source for a few seconds in a sequence.

**Figure 3:**
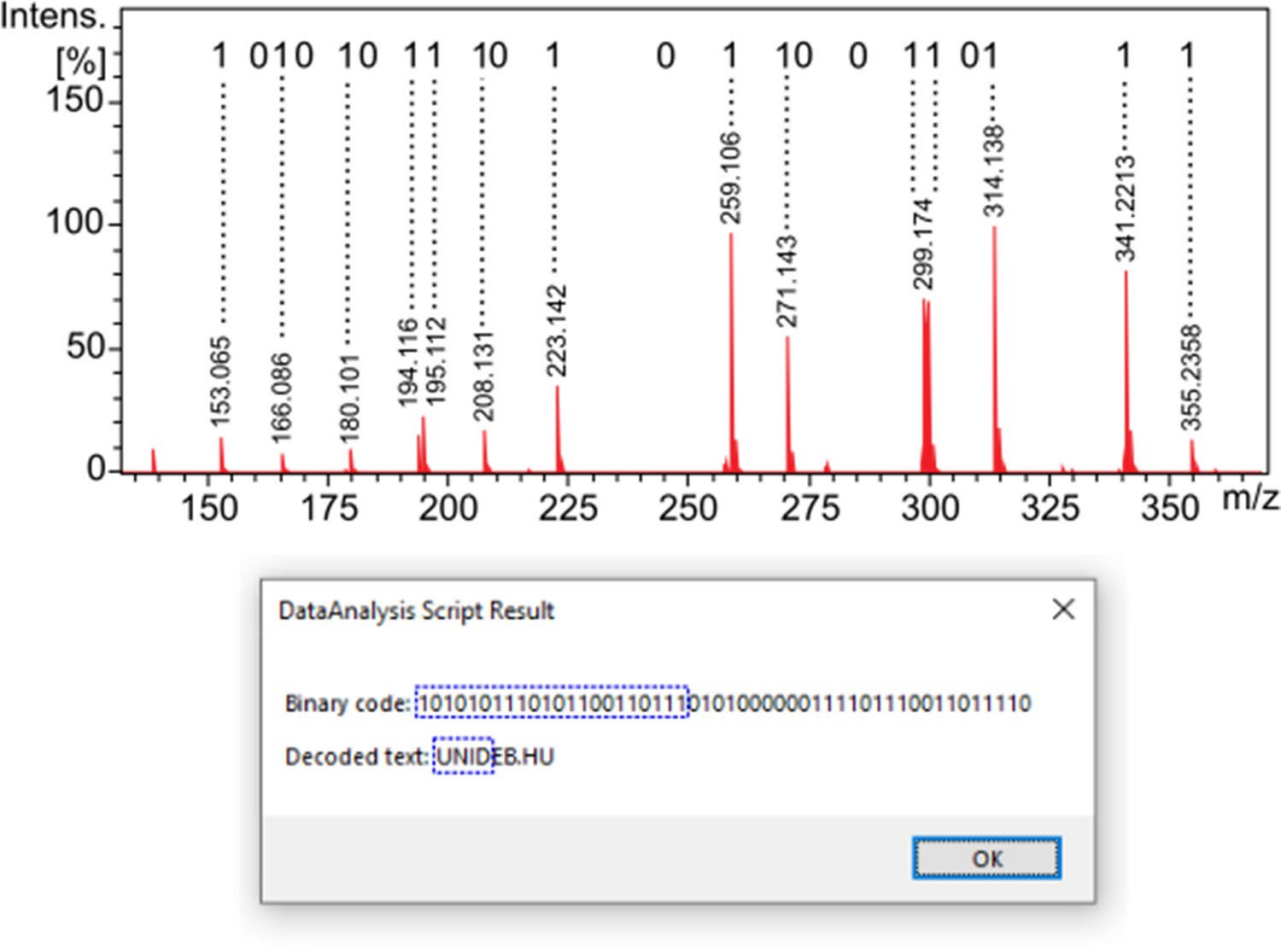
DART-MS spectrum of a solution containing a selection of nicotinic acid derivatives from the set of 22, after background subtraction. The spectrum represents 22 bits encoding the first four characters of the web link “UNIDEB.HU”. Message box (bottom) displayed by our homemade software module showing the result of the decoding.

Figure 3 shows the DART-MS spectrum of the first mixture solution, which represents the first four characters of "UNIDEB.HU", as indicated in the figure. As seen in the figure, the presence or absence of mass peaks representing individual bits can be easily and unambiguously determined, even in the case of manual evaluation. In order to speed up the decoding process and make it less subjective, we developed a macro for automated evaluation in the Bruker DataAnalysis software in Visual Basic Script language, which is reported in the Supporting Information as Algorithm S1. As conditions for accepting the presence of a peak/component, (i) its *m/z* value must not deviate by more than 0.005 from the theoretical *m/z* value, (ii) its signal-to-noise ratio must be at least 100, and (iii) its intensity must be at least one hundredth of that of the base peak. In case the peak is the second member of an *m/z* neighbor pair (see Fig. 2b and d), acceptance is subject to an additional condition, namely that (iv) its intensity must be at least a quarter of that of the previous peak. (This ensures that the isotopic peaks do not cause a false "1" bit.) The macro sequentially processes the mass spectra of the three mixture solutions, determines the binary code and decodes them into alphanumeric characters. The result (binary and text) is reported in a message box, as shown in Fig. 3. The robustness of our method is also demonstrated by the fact that the automatic evaluation gives correct results even when run before the DART background spectrum is subtracted.

**Figure 4:**
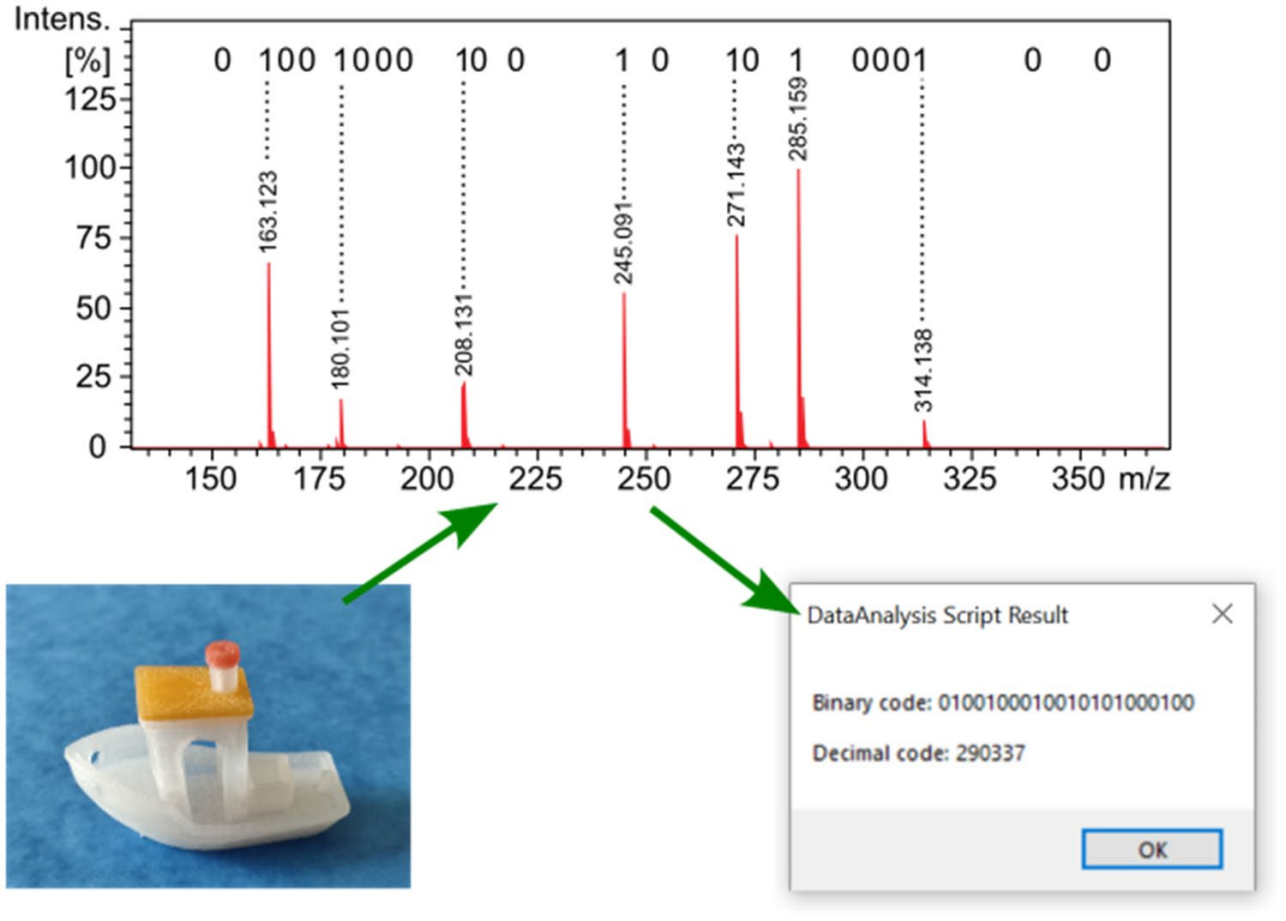
Plastic toy ship made by 3D printing, the molecular barcode is incorporated into the polypropylene matrix of the red chimney cap (bottom left). DART-MS spectrum of the red chimney cap after background subtraction. The spectrum represents 22 bits encoding the decimal barcode “290337”. Message box displayed by our homemade software module showing the result of the decoding (bottom right).

Our second proof-of-principle application, we think, is even more interesting. A numerical code, which can act as a barcode, is incorporated into the material of an object, such as a commercial product. As seen in Fig. 4, a plastic toy ship was made by 3D printing. The 22-bit nicotinic acid mixture solution was mixed with the polypropylene matrix of the red chimney cap (as detailed in the “Experimental” Section). The first 20 bits out of 22 encode the arbitrary 6-digit decimal barcode "290337" using the numeric mode of the ISO/IEC 18004 QR Code standard^23^.

As seen in Fig. 4, the presence of individual nicotinic acid components, which represent bits with a value of 1, can be clearly identified in the spectrum. In this case, we also wrote an evaluation, decoding macro in Bruker's DataAnalysis (see in the Supporting Information as Algorithm S2). This script uses the same conditions as the one above for the extraction of the bits from the mass spectrum.

## Conclusions

In this study, nicotine acid derivatives were synthesized and used to chemically encode information. The digital data are stored in mixture of solutions as the presence or absence of the derivatives. Moreover, these derivatives can be built into the matrix material of common items, as it was demonstrated in this paper. For read-out, an ambient, non-destructive method, the DART-MS was used, which can ionize molecules directly from the surface of ordinary objects. Our hypothesis that nicotinic acid derivatives would be ideal molecules for DART detection was confirmed, as the components representing the bits dominated the DART-MS spectra when the stored information was read back. The high intensity of the "bit mass peaks" ensured error-free decoding and enabled software automation of mass spectrum evaluation and decoding. Perfect read-back was achieved even though we used six components whose *m/z* values coincide with the first isotopic peak of another component. In this case, by adjusting the concentration ratios of the components during solution preparation, the presence/absence of these peaks could be clearly determined. Of course, our method can be made even more robust by omitting the six overlapping components. This leaves us with 16 components as bits, which is even more compliant with digital data storage standards. The resolution of the mass spectrometer used for the reading is relatively low, 8000 (FWHM), while the reading method was robust. A higher resolution allows the reliable detection of numerous compounds simultaneously, resulting in a higher bit density. In addition, our work demonstrated that the polymer processing methods, such as mixing with the polymer matrix, granulation, filament extrusion, 3D printing, did not affect the DART-MS detectability of the nicotinic acid compounds. Increasing the volume and density of molecular data storage was not the aim of our method. Our preliminary experiments have provided the basis for a method to incorporate a barcode or QR code into the material and surface of various objects, especially made of plastic. These are relatively short digital codes that can be used, for example, for product identification or anti-counterfeiting information.

These embedded QR codes are limited in their ability to store large amounts of data from high-resolution mass spectrometry (typical mass range *m*/*z* 50–950), metabolomics, lipidomics or proteomics.

### Supplementary Information

Supplementary Information.

## Data Availability

All data generated or analysed during this study are included in this published article and its supplementary information files.
