# Supplementary material for: Molecular data storage using direct analysis in real time (DART) ionization mass spectrometry for decoding
Source: Sci Rep. 2023 Oct 3;13:16576. doi: 10.1038/s41598-023-43658-x (PMC10547761; doi:10.1038/s41598-023-43658-x)
Supplement: Supplementary file 1 — Supplementary Information. [file 41598_2023_43658_MOESM1_ESM.docx]

**Molecular data storage using direct analysis in real time (DART) ionization mass spectrometry for decoding**

Veronika Pardi-Tóth^1,2^, Ákos Kuki ^1^, Marcell Kordován^1,2^, Gergő Róth^1,2^, Lajos Nagy^1^,
Miklós Zsuga^1^, Tibor Nagy ^1*^, Sándor Kéki^1,^

*^1^Department of Applied Chemistry, Faculty of Science and Technology, University of Debrecen, Egyetem tér 1, H-4032 Debrecen, Hungary*

*^2^Doctoral School of Chemistry, University of Debrecen, Egyetem tér 1, H-4032 Debrecen, Hungary*

***Characterization of the nicotinic acid derivatives***

**3-(1-Methyl-pyrrolidin-2-yl)-pyridine** C_10_H_14_N_2_ [M/Z]^+^_cal_ 163.123 ^1^H NMR δ 8.54 (d, *J* = 2.3 Hz, 1H), 8.50 (dd, *J* = 4.8, 1.7 Hz, 1H), 7.71 (dt, *J* = 7.9, 2.0 Hz, 1H), 7.29 – 7.22 (m, 1H), 3.26 (t, *J* = 8.3 Hz, 1H), 3.09 (t, *J* = 8.3 Hz, 1H), 2.39 – 2.24 (m, 2H), 2.18 (d, *J* = 1.2 Hz, 3H), 2.06 – 1.94 (m, 1H), 1.73 (d, *J* = 28.7 Hz, 2H).

**6-[(Benzyl-methyl-amino)-methyl]-nicotinic acid methyl ester (3a).** C_16_H_18_N_2_O_2_ [M/Z]^+^_cal_ 271.144 ^1^H NMR δ 9.18 (d, *J* = 2.2 Hz, 1H), 8.32 (dd, *J* = 8.1, 2.2 Hz, 1H), 7.68 (d, *J* = 8.2 Hz, 1H), 7.43 (s, 2H), 7.38 (s, 3H), 3.99 (s, 2H), 3.96 (s, 3H), 3.91 (s, 2H), 2.47 (s, 3H).

**4-Amino-nicotinic acid methyl ester (2a).** C_7_H_8_N_2_O_2_ [M/Z]^+^_cal_ 153.066 ^1^H NMR δ 8.90 (s, 1H), 8.20 (d, *J* = 5.9 Hz, 1H), 6.51 (d, *J* = 5.8 Hz, 1H), 6.16 (s, 2H), 3.91 (s, 3H)

**2-(Pyridin-3-yloxy)-nicotinic acid ethyl ester** **(4a).** C_13_H_12_N_2_O_3_ [M/Z]^+^_cal_ 245.092 ^1^H NMR δ 8.53 (d, *J* = 2.7 Hz, 1H), 8.49 (dd, *J* = 4.7, 1.5 Hz, 1H), 8.30 (dd, *J* = 7.5, 2.0 Hz, 1H), 8.25 (dd, *J* = 4.9, 2.0 Hz, 1H), 7.56 (ddd, *J* = 8.3, 2.7, 1.4 Hz, 1H), 7.38 (dd, *J* = 8.3, 4.8 Hz, 1H), 7.13 (dd, *J* = 7.6, 4.9 Hz, 1H), 4.42 (qd, *J* = 7.1, 6.3, 1.8 Hz, 2H), 1.40 (td, *J* = 7.2, 1.5 Hz, 3H).

**6-[(Benzyl-methyl-amino)-methyl]-nicotinic acid ethyl ester (3b).** C_17_H_20_N_2_O_2_ [M/Z]^+^_cal_ 285.159 ^1^H NMR δ 9.25 (d, *J* = 2.2 Hz, 1H), 8.39 (dd, *J* = 8.1, 2.2 Hz, 1H), 7.76 (d, *J* = 8.1 Hz, 1H), 7.60 – 7.52 (m, 2H), 7.45 (dt, *J* = 3.8, 2.4 Hz, 3H), 4.43 (dd, *J* = 7.0, 3.5 Hz, 4H), 4.17 (q, *J* = 7.1 Hz, 2H), 2.84 (s, 3H), 1.42 (t, *J* = 7.1 Hz, 3H).

**Nicotinic acid propyl ester** **(1a).** C_9_H_11_NO_2_ [M/Z]^+^_cal_ 166.086 ^1^H NMR δ 9.25 (d, *J* = 2.5 Hz, 1H), 8.81 (dd, *J* = 4.8, 1.9 Hz, 1H), 8.34 (dt, *J* = 8.0, 2.0 Hz, 1H), 7.44 (dd, *J* = 8.0, 4.9 Hz, 1H), 4.33 (td, *J* = 6.7, 1.7 Hz, 2H), 1.81 (qd, *J* = 9.0, 8.1, 6.3 Hz, 2H), 1.04 (td, *J* = 7.4, 1.8 Hz, 3H).

**5-(3-Formyl-4-methoxy-phenyl)-nicotinic acid propyl ester** **(5a).** C_17_H_17_NO_4_ [M/Z]^+^_cal_ 300.123 ^1^H NMR δ 10.54 (s, 1H), 9.19 (d, *J* = 2.1 Hz, 1H), 8.98 (d, *J* = 2.4 Hz, 1H), 8.47 (t, *J* = 2.2 Hz, 1H), 8.12 (d, *J* = 2.6 Hz, 1H), 7.83 (dd, *J* = 8.7, 2.6 Hz, 1H), 7.15 (d, *J* = 8.7 Hz, 1H), 4.36 (t, *J* = 6.7 Hz, 2H), 4.02 (s, 3H), 1.84 (h, *J* = 7.2 Hz, 2H), 1.05 (t, *J* = 7.4 Hz, 3H).

**2-(Pyridin-3-yloxy)-nicotinic acid propyl ester** **(4b).** C_14_H_14_N_2_O_3_ [M/Z]^+^_cal_ 259.108 ^1^H NMR δ 8.51 (d, *J* = 2.8 Hz, 1H), 8.49 – 8.46 (m, 1H), 8.32 – 8.22 (m, 2H), 7.53 (d, *J* = 8.3 Hz, 1H), 7.36 (dd, *J* = 8.3, 4.6 Hz, 1H), 7.12 (dd, *J* = 7.6, 4.7 Hz, 1H), 4.33 (t, *J* = 6.6 Hz, 2H), 1.79 (q, *J* = 7.1 Hz, 2H), 1.02 (t, *J* = 7.4 Hz, 3H).

**2-(Pyridin-3-yloxy)-nicotinic acid butyl ester** **(4c).** C_15_H_16_N_2_O_3_ [M/Z]^+^_cal_ 273.123 ^1^H NMR δ 8.51 (s, 1H), 8.47 (d, *J* = 4.8 Hz, 1H), 8.27 (dd, *J* = 13.3, 6.1 Hz, 2H), 7.53 (d, *J* = 8.3 Hz, 1H), 7.36 (dd, *J* = 8.4, 4.7 Hz, 1H), 7.12 (dd, *J* = 7.6, 4.8 Hz, 1H), 4.37 (t, *J* = 6.5 Hz, 2H), 1.78 – 1.72 (m, 2H), 1.50 – 1.43 (m, 2H), 0.96 (t, *J* = 7.4 Hz, 3H).

**Nicotinic acid butyl ester** **(1b).** C_10_H_13_NO_2_ [M/Z]^+^_cal_ 180.102 ^1^H NMR δ 9.23 (d, *J* = 2.4 Hz, 1H), 8.78 (dd, *J* = 4.9, 1.8 Hz, 1H), 8.31 (dt, *J* = 8.0, 2.0 Hz, 1H), 7.40 (dd, *J* = 8.0, 4.9 Hz, 1H), 4.37 (t, *J* = 6.6 Hz, 2H), 1.77 (p, *J* = 6.8 Hz, 2H), 1.55 – 1.42 (m, 2H), 0.99 (t, *J* = 7.4 Hz, 3H).

**Nicotinic acid hexyl ester** **(1d).** C_12_H_17_NO_2_ [M/Z]^+^_cal_ 208.133 ^1^H NMR δ 9.23 (d, *J* = 2.2 Hz, 1H), 8.78 (dd, *J* = 4.9, 1.7 Hz, 1H), 8.31 (dt, *J* = 8.0, 2.0 Hz, 1H), 7.40 (dd, *J* = 8.0, 4.9 Hz, 1H), 4.36 (t, *J* = 6.7 Hz, 2H), 1.78 (p, *J* = 6.9 Hz, 2H), 1.35 (tt, *J* = 8.6, 4.4 Hz, 6H), 0.94 – 0.89 (m, 3H).

**6-[(Benzyl-methyl-amino)-methyl]-nicotinic acid propyl ester** **(3c).** C_18_H_22_N_2_O_2_ [M/Z]^+^_cal_ 299.175 ^1^H NMR δ 9.24 – 9.05 (m, 1H), 8.28 (dd, *J* = 8.2, 2.2 Hz, 1H), 7.64 (d, *J* = 8.1 Hz, 1H), 7.49 – 7.16 (m, 5H), 4.31 (t, *J* = 6.7 Hz, 2H), 3.76 (s, 2H), 3.62 (s, 2H), 2.27 (s, 3H), 1.81 (h, *J* = 7.1 Hz, 2H), 1.03 (t, *J* = 7.4 Hz, 3H).

**6-[(Benzyl-methyl-amino)-methyl]-nicotinic acid butyl ester** **(3d).** C_19_H_24_N_2_O_2_ [M/Z]^+^_cal_ 313.191 ^1^H NMR δ 9.14 (d, *J* = 2.2 Hz, 1H), 8.27 (dd, *J* = 8.1, 2.2 Hz, 1H), 7.64 (d, *J* = 8.2 Hz, 1H), 7.41 – 7.25 (m, 5H), 4.35 (t, *J* = 6.6 Hz, 2H), 3.78 (s, 2H), 3.63 (s, 2H), 2.28 (s, 3H), 1.79 – 1.71 (m, 2H), 1.52 – 1.43 (m, 2H), 0.98 (t, *J* = 7.4 Hz, 3H).

**5-(3-Formyl-4-methoxy-phenyl)-nicotinic acid butyl ester** **(5b).** C_18_H_19_NO_2_ [M/Z]^+^_cal_ 314.139 ^1^H NMR δ 10.54 (s, 1H), 9.18 (d, *J* = 2.0 Hz, 1H), 8.99 (d, *J* = 2.4 Hz, 1H), 8.47 (t, *J* = 2.2 Hz, 1H), 8.12 (d, *J* = 2.6 Hz, 1H), 7.84 (dd, *J* = 8.7, 2.6 Hz, 1H), 7.16 (d, *J* = 8.7 Hz, 1H), 4.40 (t, *J* = 6.6 Hz, 2H), 4.02 (s, 3H), 1.79 (q, *J* = 7.2 Hz, 2H), 1.50 (q, *J* = 7.5 Hz, 2H), 1.00 (t, *J* = 7.4 Hz, 3H).

**6-[(Benzyl-methyl-amino)-methyl]-nicotinic acid hexyl ester (3e).** C_21_H_28_N_2_O_2_ [M/Z]^+^_cal_ 341.222 ^1^H NMR δ 9.21 (d, *J* = 2.1 Hz, 1H), 8.35 (dd, *J* = 8.2, 2.2 Hz, 1H), 7.71 (d, *J* = 8.1 Hz, 1H), 7.49 – 7.29 (m, 5H), 4.41 (t, *J* = 6.7 Hz, 2H), 3.83 (s, 2H), 3.68 (s, 2H), 2.34 (s, 3H), 1.84 (p, *J* = 6.8 Hz, 2H), 1.51 (t, *J* = 7.3 Hz, 2H), 1.41 (t, *J* = 3.8 Hz, 2H), 1.30 (q, *J* = 7.3 Hz, 2H), 0.98 (q, *J* = 5.4 Hz, 3H).

**6-[(Benzyl-methyl-amino)-methyl]-nicotinic acid heptyl ester** **(3f).** C_22_H_30_N_2_O_2_ [M/Z]^+^_cal_ 355.238 ^1^H NMR δ 9.14 (d, *J* = 2.2 Hz, 1H), 8.27 (dd, *J* = 8.2, 2.2 Hz, 1H), 7.64 (d, *J* = 8.2 Hz, 1H), 7.46 – 7.19 (m, 5H), 4.34 (t, *J* = 6.7 Hz, 2H), 3.76 (s, 2H), 3.68 (s, 2H), 2.27 (s, 3H), 1.77 (p, *J* = 6.7 Hz, 2H), 1.58 (q, *J* = 6.9 Hz, 2H), 1.30 (qd, *J* = 12.3, 11.6, 5.4 Hz, 6H), 0.89 (t, *J* = 6.7 Hz, 3H).

**4-Amino-nicotinic acid ethyl ester** **(2b).** C_8_H_10_N_2_O_2_ [M/Z]^+^_cal_ 167.087 ^1^H NMR δ 8.91 (s, 1H), 8.20 (d, *J* = 5.9 Hz, 1H), 6.52 (d, *J* = 5.9 Hz, 1H), 4.37 (q, *J* = 7.1 Hz, 2H), 1.40 (t, *J* = 7.1 Hz, 3H).

**4-Amino-nicotinic acid propyl ester** **(2c).** C_9_H_12_N_2_O_2_ [M/Z]^+^_cal_ 181.097 ^1^H NMR δ 8.92 (s, 1H), 8.20 (d, *J* = 5.9 Hz, 1H), 6.51 (d, *J* = 5.9 Hz, 1H), 6.19 (s, 2H), 4.27 (t, *J* = 6.6 Hz, 2H), 1.83 – 1.75 (m, 2H), 1.04 (t, *J* = 7.4 Hz, 3H).

**4-Amino-nicotinic acid butyl ester** **(2d).** C_10_H_14_N_2_O_2_ [M/Z]^+^_cal_ 195.113 ^1^H NMR δ 8.91 (s, 1H), 8.19 (d, *J* = 5.9 Hz, 1H), 6.52 (d, *J* = 5.9 Hz, 1H), 6.29 (s, 2H), 4.31 (t, *J* = 6.6 Hz, 2H), 1.76 (p, *J* = 6.9 Hz, 2H), 1.56 – 1.40 (m, 2H), 0.96 (dt, *J* = 16.0, 7.3 Hz, 3H).

**4-Amino-nicotinic acid hexyl ester** **(2f).** C_12_H_18_N_2_O_2_ [M/Z]^+^_cal_ 223.144 ^1^H NMR δ 8.92 (s, 1H), 8.23 (d, *J* = 6.2 Hz, 1H), 6.85 (d, *J* = 6.1 Hz, 1H), 4.40 (dt, *J* = 17.3, 6.7 Hz, 2H), 4.15 (dt, *J* = 10.8, 6.8 Hz, 2H), 1.53 – 1.28 (m, 6H), 1.04 – 0.88 (m, 3H)

**Nicotinic acid pentyl ester** **(1c).** C_11_H_15_NO_2_ [M/Z]^+^_cal_ 194.118 ^1^H NMR δ 9.31 (d, *J* = 2.2 Hz, 1H), 8.87 (dd, *J* = 4.9, 1.7 Hz, 1H), 8.41 (dt, *J* = 8.0, 2.0 Hz, 1H), 7.51 (dd, *J* = 7.9, 4.9 Hz, 1H), 4.43 (t, *J* = 6.7 Hz, 2H), 1.86 (p, *J* = 6.6 Hz, 2H), 1.49 (dt, *J* = 6.8, 3.1 Hz, 4H), 1.01 (t, *J* = 6.9 Hz, 3H).

**4-Amino-nicotinic acid pentyl ester** **(2e).** C_11_H_16_N_2_O_2_ [M/Z]^+^_cal_ 209.128 ^1^H NMR δ 8.61 (s, 1H), 7.99 (d, *J* = 7.2 Hz, 1H), 7.36 (d, *J* = 7.2 Hz, 1H), 4.33 (t, *J* = 6.7 Hz, 2H), 4.10 (t, *J* = 6.7 Hz, 2H), 1.38 (dt, *J* = 6.7, 3.4 Hz, 4H), 0.91 (dt, *J* = 13.7, 4.8 Hz, 3H).

**Table S1.** Concentrations of the nicotinic acid derivatives as molecular bits in the polypropylene matrix used for the 3D printing. In our example, the components marked with an asterisk represent bits with a value of 1.

| Compound | [M+H]^+^  *m/z* | m/m % | ppm |
| --- | --- | --- | --- |
| **2a** | 153.066 | 0.026 | 260 |
| **Nicotine*** | 163.123 | 0.127 | 1266 |
| **1a** | 166.086 | 0.402 | 4016 |
| **2b** | 167.082 | 0.017 | 167 |
| **1b*** | 180.102 | 0.260 | 2604 |
| **2c** | 181.097 | 0.172 | 1718 |
| **1c** | 194.118 | 0.232 | 2318 |
| **2d** | 195.113 | 0.025 | 251 |
| **1d*** | 208.133 | 0.118 | 1185 |
| **2e** | 209.128 | 0.030 | 303 |
| **2f** | 223.144 | 0.112 | 1117 |
| **4a*** | 245.092 | 0.072 | 715 |
| **4b** | 259.108 | 0.055 | 552 |
| **3a*** | 271.144 | 0.016 | 158 |
| **4c** | 273.123 | 0.124 | 1244 |
| **3b*** | 285.159 | 0.021 | 210 |
| **3c** | 299.175 | 0.025 | 245 |
| **5a** | 300.123 | 0.129 | 1285 |
| **3d** | 313.191 | 0.025 | 252 |
| **5b*** | 314.139 | 0.131 | 1309 |
| **3e** | 341.222 | 0.085 | 847 |
| **3f** | 355.238 | 0.188 | 1883 |

**Table S2.** Suggested concentrations of the nicotinic acid derivatives

| Compound | [M+H]^+^  *m/z* | Concentration  mg/mL |
| --- | --- | --- |
| **2a** | 153.066 | 0.16 |
| **Nicotine** | 163.123 | 0.02 |
| **1a** | 166.086 | 0.11 |
| **2b** | 167.082 | 0.12 |
| **1b** | 180.102 | 0.11 |
| **2c** | 181.097 | 0.16 |
| **1c** | 194.118 | 0.17 |
| **2d** | 195.113 | 0.10 |
| **1d** | 208.133 | 0.13 |
| **2e** | 209.128 | 0.02 |
| **2f** | 223.144 | 0.22 |
| **4a** | 245.092 | 0.18 |
| **4b** | 259.108 | 0.44 |
| **3a** | 271.144 | 0.02 |
| **4c** | 273.123 | 0.45 |
| **3b** | 285.159 | 0.04 |
| **3c** | 299.175 | 0.02 |
| **5a** | 300.123 | 1.81 |
| **3d** | 313.191 | 0.01 |
| **5b** | 314.139 | 1.72 |
| **3e** | 341.222 | 0.03 |
| **3f** | 355.238 | 0.01 |

**Algorithm S1.** Visual Basic Script macro for decoding the MS spectra of the first proof-of-principle application

Set fs = CreateObject("Scripting.FileSystemObject")

Set a = fs.CreateTextFile("results.txt", True)

Dim Peak, Spect, PL, bits(23), mzbit, PrevInt, dec1, codes(10), Sdecoded, Sbits

mzbits = array(,153.066,163.123,166.086,167.082,180.102,181.097,194.118, _

195.113,208.133,209.128,223.144,245.092,259.108,271.144,273.123,285.159, _

299.175,300.123,313.191,314.139,341.222,355.238)

Sbits=""

set Spect = Application.Analyses.Item(1).Spectra(1)

ReadBits(22)

dec1=0

for i=0 to 10

dec1=dec1+bits(11-i)*2^i

next

dec2=0

for i=0 to 10

dec2=dec2+bits(22-i)*2^i

next

codes(1)=dec1\45

codes(2)=dec1 mod 45

codes(3)=dec2\45

codes(4)=dec2 mod 45

set Spect = Application.Analyses.Item(1).Spectra(2)

ReadBits(22)

dec1=0

for i=0 to 10

dec1=dec1+bits(11-i)*2^i

next

dec2=0

for i=0 to 10

dec2=dec2+bits(22-i)*2^i

next

codes(5)=dec1\45

codes(6)=dec1 mod 45

codes(7)=dec2\45

codes(8)=dec2 mod 45

set Spect = Application.Analyses.Item(1).Spectra(3)

ReadBits(6)

dec1=0

for i=0 to 5

dec1=dec1+bits(6-i)*2^i

next

codes(9)=dec1

Sdecoded=""

for i=1 to 9

if codes(i)<10 then

Sdecoded=Sdecoded+chr(codes(i)+48)

else

if codes(i)<36 then Sdecoded=Sdecoded+chr(codes(i)+55) end if

end if

Select Case codes(i)

Case 36

Sdecoded=Sdecoded+" "

Case 37

Sdecoded=Sdecoded+"$"

Case 38

Sdecoded=Sdecoded+"%"

Case 39

Sdecoded=Sdecoded+"*"

Case 40

Sdecoded=Sdecoded+"+"

Case 41

Sdecoded=Sdecoded+"-"

Case 42

Sdecoded=Sdecoded+"."

Case 43

Sdecoded=Sdecoded+"/"

Case 44

Sdecoded=Sdecoded+":"

End Select

next

a.WriteLine(Sbits)

a.WriteLine(Sdecoded)

Msgbox "Binary code: "+Sbits+chr(13)+chr(13)+"Decoded text: "+Sdecoded,0,"DataAnalysis Script Result"

a.Close

form.close

sub ReadBits (nbits)

set PL = Spect.MSPeakList

maxint=Spect.MaximumIntensity

For mzbit=1 to nbits

bits(mzbit)=0

PrevInt=0

For Each Peak in PL

if Peak.SignalToNoise>100 and _

mzbits(mzbit)-0.005<Peak.m_over_z and mzbits(mzbit)+0.005>Peak.m_over_z _

and Peak.Intensity>maxInt/100 then

bits(mzbit)=1

if (mzbit=4 or mzbit=6 or mzbit=8 or mzbit=10 or mzbit=15 or mzbit=18 or mzbit=20) _

and Peak.Intensity<PrevInt/4 then bits(mzbit)=0

end if

PrevInt=Peak.Intensity

next

Sbits=Sbits+chr(bits(mzbit)+48)

Next

end sub

**Algorithm S2.** Visual Basic Script macro for decoding the MS spectrum of the second proof-of-principle application

Set fs = CreateObject("Scripting.FileSystemObject")

Set a = fs.CreateTextFile("results.txt", True)

Dim Peak, Spect, PL, bits(23), mzbit, PrevInt, dec1, codes(9), Sdecoded, Sbits

mzbits = array(,153.066,163.123,166.086,167.082,180.102,181.097,194.118, _

195.113,208.133,209.128,223.144,245.092,259.108,271.144,273.123,285.159, _

299.175,300.123,313.191,314.139,341.222,355.238)

Sbits=""

set Spect = Application.Analyses.Item(1).Spectra(1)

set PL = Spect.MSPeakList

maxint=Spect.MaximumIntensity

For mzbit=1 to 22

bits(mzbit)=0

PrevInt=0

For Each Peak in PL

if Peak.SignalToNoise>10 and _

mzbits(mzbit)-0.005<Peak.m_over_z and mzbits(mzbit)+0.005>Peak.m_over_z and _

Peak.Intensity>PrevInt/4 and Peak.Intensity>maxInt/100 then _

bits(mzbit)=1

PrevInt=Peak.Intensity

next

Sbits=Sbits+chr(bits(mzbit)+48)

Next

dec1=0

for i=0 to 9

dec1=dec1+bits(10-i)*2^i

next

dec2=0

for i=0 to 9

dec2=dec2+bits(20-i)*2^i

next

Sdecoded=CStr(dec1 \ 100)+CStr((dec1 mod 100) \10)+CStr(dec1 mod 10)+CStr(dec2 \ 100)+ _

CStr((dec2 mod 100) \10)+CStr(dec2 mod 10)

a.WriteLine(Sbits)

a.WriteLine(Sdecoded)

Msgbox "Binary code: "+Sbits+chr(13)+chr(13)+"Decimal code: "+Sdecoded,0,"DataAnalysis Script Result"

a.Close

form.close
